# In Vivo Secretion of β-Lactamase-Carrying Outer Membrane Vesicles as a Mechanism of β-Lactam Therapy Failure

**DOI:** 10.3390/membranes11110806

**Published:** 2021-10-23

**Authors:** Martina Bielaszewska, Ondřej Daniel, Otakar Nyč, Alexander Mellmann

**Affiliations:** 1 Centre for Epidemiology and Microbiology, National Institute of Public Health, 100 00 Prague, Czech Republic; ondrej.daniel@szu.cz; 2 Second Medical Faculty, Charles University, 150 06 Prague, Czech Republic; 3 Institute of Medical Microbiology, University Hospital Motol, 150 06 Prague, Czech Republic; Otakar.Nyc@fnmotol.cz; 4 Institute for Hygiene, University Hospital Münster, 48149 Münster, Germany; alexander.mellmann@ukmuenster.de

**Keywords:** group A *Streptococcus pyogenes* (GAS), pharyngotonsillitis, amoxicillin therapy failure, β-lactamase-carrying outer membrane vesicles, *Haemophilus influenzae*, in vivo secretion, GAS protection

## Abstract

Outer membrane vesicles carrying β-lactamase (βLOMVs) protect bacteria against β-lactam antibiotics under experimental conditions, but their protective role during a patient’s treatment leading to the therapy failure is unknown. We investigated the role of βLOMVs in amoxicillin therapy failure in a patient with group A *Streptococcus pyogenes* (GAS) pharyngotonsillitis. The patient’s throat culture was examined by standard microbiological procedures. Bacterial vesicles were analyzed for β-lactamase by immunoblot and the nitrocefin assay, and in vivo secretion of βLOMVs was detected by electron microscopy. These analyses demonstrated that the patient’s throat culture grew, besides amoxicillin-susceptible GAS, an amoxicillin-resistant nontypeable *Haemophilus influenzae* (NTHi), which secreted βLOMVs. Secretion and β-lactamase activity of NTHi βLOMVs were induced by amoxicillin concentrations reached in the tonsils during therapy. The presence of NTHi βLOMVs significantly increased the minimal inhibitory concentration of amoxicillin for GAS and thereby protected GAS against bactericidal concentrations of amoxicillin. NTHi βLOMVs were identified in the patient’s pharyngotonsillar swabs and saliva, demonstrating their secretion in vivo at the site of infection. We conclude that the pathogen protection via βLOMVs secreted by the flora colonizing the infection site represents a yet underestimated mechanism of β-lactam therapy failure that warrants attention in clinical studies.

## 1. Introduction

Group A *Streptococcus pyogenes* (GAS) is the most common cause of acute bacterial pharyngotonsillitis, accounting for 20–30% of cases in children and 5–15% of cases in adults [1]. GAS is highly susceptible to β-lactam antibiotics, so penicillin and amoxicillin are the treatments of choice [1]. However, the inabilities of these antibiotics to eradicate GAS from patients with pharyngotonsillitis have been increasingly reported [2]. One cause of the therapy failure is colonization of the pharynx and tonsils by β-lactamase-producing bacteria such as *Moraxella catarrhalis*, *Haemophilus influenzae*, and *Staphylococcus aureus* that protect GAS against β-lactam antibiotics [2]. Yet, the mechanisms of this protection are incompletely understood. Here, we investigated the involvement of bacterial outer membrane vesicles (OMVs) in amoxicillin therapy failure. 

OMVs are bilayered spherical nanoparticles in size between 10 and 300 nm produced by Gram-negative bacteria [3,4,5,6]. They are formed by a bulging of the bacterial outer membrane followed by fission of the nascent vesicle and its subsequent release from the bacterial cell [6,7]. They mainly contain the outer membrane components and periplasmic proteins [3,4,5,6]. OMVs represent a novel, highly efficient bacterial secretion system [5], which mediates multiple interbacterial and microbe–host interactions [3,5,6,8,9,10]. They are produced both in vitro and in vivo [3,10,11,12]. Secretion of OMVs is significantly increased under environmental stress conditions, including those encountered by the bacteria within the host, and enables bacteria to cope with and adapt to this stress [8,13,14,15]. Notably, exposure of bacteria to particular antibiotics results in increased OMV production [16,17,18,19], suggesting that OMVs are involved in the bacterial protection against these drugs. Several mechanisms of the protection have been proposed based on experimental data [18,19,20,21,22,23,24,25,26]. One of these mechanisms, which occurs in β-lactamase-producing bacteria, involves production of OMVs that carry various β-lactamases, thereby protecting susceptible bacteria of the same and other species against β-lactam antibiotics under in vitro conditions [18,20,22,23,24,25,26]. However, the involvement of this mechanism in β-lactam therapy failure in vivo during patients´ treatment remains unknown. We therefore explored the role of β-lactamase-carrying OMVs secreted in vivo by amoxicillin-resistant *Haemophilus influenzae* in amoxicillin therapy failure in a patient with GAS pharyngotonsillitis. We demonstrated that these OMVs protected GAS against amoxicillin, which resulted in bacteriological and clinical treatment failure. 

## 2. Materials and Methods

### 2.1. Patient and Microbiological Examinations

A 39-year-old man presented with acute pharyngotonsillitis accompanied by a fever up to 39.8 °C. His throat swabs were cultured on blood agar and chocolate agar (Thermo Fisher Scientific, Prague, Czech Republic) and the isolates were identified by standard bacteriological procedures [27] and MALDI-TOF mass spectrometry (Microflex LT, Bruker Daltonics, Bremen, Germany). Antimicrobial susceptibilities were determined by the disc diffusion method (discs from Oxoid, Brno, Czech Republic) and the broth microdilution method according to the Clinical and Laboratory Standards Institute (CLSI) procedures and criteria [28]. The *bla*_TEM-1_ gene was detected by PCR followed by digestion of 600 bp amplicon with *Mbo*I (New England Biolabs, Frankfurt am Main, Germany) as described previously [29].

### 2.2. Ethical Approval

The study was conducted according to the guidelines of the Declaration of Helsinki and approved by the Ethics Committee of the National Institute of Public Health, Prague (protocol code EK-SZU/08076/2021), on 1 June 2021. Written informed consent was obtained from the patient.

### 2.3. Isolation and Characterization of OMVs Produced by Nontypeable Haemophilus Influenzae (NTHi) patient’s Isolate

The NTHi patient’s isolate was cultured overnight in a brain heart infusion (BHI) broth (Thermo Fisher Scientific, Prague, Czech Republic) supplemented with NAD (nicotinamide adenine dinucleotide) and hemin (10 µg/mL each) (Sigma-Aldrich, Taufkirchen, Germany). To determine the effect of amoxicillin on OMV production, the medium was supplemented with amoxicillin (Sigma-Aldrich, Taufkirchen, Germany) in concentrations reported in the tonsillar tissue during amoxicillin therapy (0.17 µg/mL, 1.1 µg/mL, and 3.9 µg/mL) [30,31,32]. OMVs were isolated by ultracentrifugation as described previously [33,34]. OMV amounts were determined by nanoparticle tracking analysis [15,35], morphology by electron microscopy after negative staining [34], and protein concentrations with Roti-Nanoquant (Carl Roth, Karlsruhe, Germany) in accordance with the manufacturer´s instructions. OMV-associated β-lactamase was detected by immunoblot [34] with a mouse monoclonal anti-β-lactamase antibody (Abcam, Cambridge, United Kingdom, Cat# ab12251, RRID: AB_298974) and an alkaline phosphatase-conjugated goat anti-mouse IgG (Dianova, Hamburg, Germany, Cat# 115-055-146, RRID:AB_2338538); signals were developed with NBT/BCIP (nitro blue tetrazolium chloride/5-bromo-4-chloro-3′-indolyl phosphate, toluidine salt) substrate (Roche, Mannheim, Germany) and visualized with a Chemi Doc XRS imager (BioRad, Munich, Germany). Localization of β-lactamase within OMVs was determined by the proteinase K assay, as described previously [22,36]. Briefly, OMVs, either intact or lyzed with 0.1 M EDTA (ethylenediaminetetraacetic acid disodium salt dihydrate) (Sigma-Aldrich, Taufkirchen, Germany), were treated with proteinase K (Sigma-Aldrich, Taufkirchen, Germany) (100 µg/mL, 30 min). After deactivation with PMSF (phenylmethylsulfonyl fluoride) (Roche, Mannheim, Germany) and AEBSF (4-(2- aminoethyl) benzenesulfonyl fluoride hydrocholoride) (Sigma-Aldrich, Taufkirchen, Germany), the samples were analyzed by immunoblot as described above. β-lactamase activity in OMVs was quantified with the β-lactamase activity assay kit (Sigma-Aldrich, Taufkirchen, Germany) as recommended by the manufacturer. 

### 2.4. GAS Protection against Amoxicillin via β-Lactamase-Carrying, Amoxicillin-Induced NTHi OMVs

To determine GAS protection against amoxicillin via β-lactamase-carrying, amoxicillin-induced NTHi OMVs (hereafter designated NTHi OMVs_βL+AMX+_), GAS was cultured for 24 h in BHI broth supplemented with amoxicillin in concentrations reported in the tonsils of amoxicillin-treated patients (0.17 µg/mL or 3.9 µg/mL) [30,32], and: (i) NTHi OMVs_βL+AMX+_ (724 µg/mL or 1.2 mg/mL of OMV protein); or (ii) NTHi OMVs_βL+AMX+_ (1.2 mg/mL of OMV protein) and potassium clavulanate (Sigma-Aldrich, Taufkirchen, Germany; 25 µg/mL); or (iii) β-lactamase-negative NTHi OMVs (1.2 mg/mL of OMV protein) from a control amoxicillin-susceptible NTHi from our collection (amoxicillin minimal inhibitory concentration (MIC) of 0.5 µg/mL). Growth was quantified by determination of colony-forming units (CFU)/mL. The amounts of NTHi OMVs_βL+AMX+_ used, i.e., 724 µg/mL and 1.2 mg/mL, corresponded to those produced by NTHi in the presence of amoxicillin concentrations of 0.17 µg/mL and 3.9 µg/mL, respectively (Table 1). GAS culture in BHI broth without amoxicillin and in BHI broth with amoxicillin (0.17 µg/mL or 3.9 µg/mL) but without NTHi OMVs_βL+AMX+_ served as a control of GAS growth and of amoxicillin-mediated GAS inhibition, respectively. After 24 h, each culture was streaked on BHI agar without amoxicillin and with amoxicillin (0.17 µg/mL or 3.9 µg/mL) and checked for growth after overnight incubation. 

### 2.5. The Influence of NTHi OMVs_βL+AMX+_ on Amoxicillin MIC for GAS

To determine the influence of NTHi OMVs_βL+AMX+_ on amoxicillin MIC for GAS, MIC was determined for: (i) GAS alone; (ii) GAS supplemented with NTHi OMVs_βL+AMX+_ (724 µg/mL or 1.2 mg/mL of OMV protein); (iii) GAS supplemented with NTHi OMVs_βL+AMX+_ (1.2 mg/mL of OMV protein) and potassium clavulanate (25 µg/mL); and iv) GAS supplemented with β-lactamase-negative OMVs from the control amoxicillin-susceptible NTHi (1.2 mg/mL of OMV protein).

### 2.6. In Vivo Detection of OMV-Producing H. Influenzae and β-Lactamase-Carrying OMVs

Pharyngotonsillar swabs, exudate from tonsillar crypts, and saliva were taken from the patient and processed for transmission electron microscopy as described previously [34] with minor modifications. Briefly, the pharyngotonsillar swabs and swabs soaked with crypt exudate were placed into phosphate-buffered saline (PBS) with 2% paraformaldehyde and 0.2% glutaraldehyde (Sigma-Aldrich, Taufkirchen, Germany), vigorously vortexed to release bacteria and OMVs, fixed for 30 min, ultracentrifuged (52,000× *g*, 30 min), and the pellets were embedded in 6% gelatine (fish skin gelatine, BioTrend, Miramar Beach, USA). Saliva (15 mL) was mixed with 15 mL of PBS, and vesicles were collected by ultracentrifugation and embedded in gelatine as above. Ultrathin (50 nm) cryosections were cut from the gelatine blocks (cryo-ultramicrotome Leica Ultracut EM UC7; Leica Microsystem, Wetzlar, Germany), placed on a formvar film and carbon-coated copper grid (Sigma-Aldrich, Taufkirchen, Germany), and stained with a mouse monoclonal anti-β-lactamase antibody (Abcam, Cambridge, UK, Cat# ab12251, RRID:AB_298974) and goat anti-mouse IgG conjugated with 10 nm gold (Abcam, Cambridge, UK, Cat# ab39619, RRID:AB_954440). The preparations were postfixed with glutaraldehyde (Sigma-Aldrich, Taufkirchen, Germany), contrasted with uranyl acetate (Thermo Fisher Scientific, Erlangen, Germany), and examined with an HT7800 transmission electron microscope (Hitachi High-Tech Analytical Science, Prague, Czech Republic).

### 2.7. Statistical Analysis

Data were analyzed with one-way ANOVA (analysis of variance); *p* < 0.05 was considered significant.

## 3. Results

### 3.1. Amoxicillin Treatment Failure in a Patient with GAS Pharyngotonsillitis

The throat culture of the patient with pharyngotonsillitis grew GAS, which was susceptible to penicillin, ampicillin, and amoxicillin (Table 2). The patient was treated with amoxicillin (750 mg three times daily, p.o.) for 10 days, but no improvement was observed. A repeated throat culture performed after completing the amoxicillin treatment continued growing amoxicillin-susceptible GAS. Moreover, this second culture revealed NTHi, which was resistant to amoxicillin (MIC of 16 µg/mL), carried the *bla*_TEM-1_ gene, and was likely selected by the amoxicillin therapy. It was susceptible to amoxicillin/clavulanate (Table 2). The patient was treated with amoxicillin/clavulanate (1 g twice daily, p.o.) and fully recovered after 4 days; the therapy was continued for up to 10 days. Control throat cultures performed 2 days and 10 days after termination of the amoxicillin/clavulanate therapy were negative for GAS and NTHi. 

### 3.2. NTHi patient’s Isolate Secretes β-Lactamase-Carrying OMVs That Are Induced by Amoxicillin

**Figure 1:**
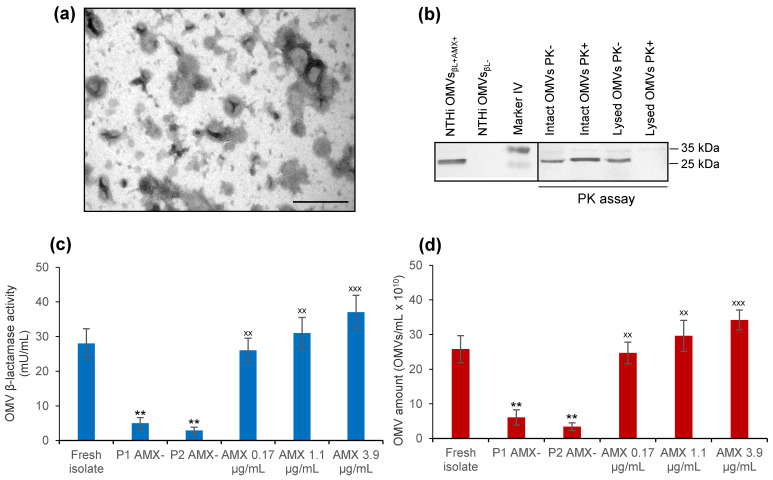
Nontypeable *Haemophilus influenzae* (NTHi) isolated from the patient’s throat culture secretes β-lactamase-carrying OMVs, which are induced by amoxicillin (NTHi OMVs_βL+AMX+_): (**a**) electron microscopy of negatively stained NTHi OMVs_βL+AMX+_; scale bar, 200 nm. (**b**) OMV immunoblot with anti-β-lactamase antibody. Left panel: lane 1, NTHi OMVs_βL+AMX+_, lane 2, control β-lactamase-negative OMVs (NTHi OMVs_βL−_), lane 3, protein size marker (the marker band sizes are on the right side); β-lactamase is ~32 kDa. Right panel: OMVs_βL+AMX+_ subjected to proteinase K (PK) assay, which demonstrates intravesicular localization of β-lactamase. (**c**,**d**) β-lactamase activities (**c**) and the amounts (**d**) of OMVs produced by NTHi freshly isolated from the patient’s throat culture (fresh isolate), by NTHi passaged twice in BHI broth without amoxicillin (P1 and P2, AMX-), and by NTHi from passage 2 grown in BHI broth with amoxicillin concentrations reported in the tonsils during therapy (0.17 µg/mL, 1.1 µg/mL, or 3.9 µg/mL). Data are presented as means ± standard deviations from three independent experiments; ** *p* < 0.01 compared to fresh isolate; ^xx^ *p* < 0.01 compared to P2 AMX-; ^xxx^ *p* < 0.001 compared to P2 AMX- (statistical analysis was performed with one-way ANOVA).

To gain insight into the role of NTHi OMVs in the amoxicillin therapy failure, we isolated OMVs from the NTHi patient’s isolate (Figure 1a) and analyzed them for the presence of β-lactamase and β-lactamase activity. The OMVs contained β-lactamase (Figure 1b), which was located inside OMVs, as demonstrated by its protection against proteinase K (PK) in the PK assay (Figure 1b). The β-lactamase was enzymatically active, as evidenced by the ability of OMVs to cleave the β-lactamase substrate nitrocefin (Figure 1c). Notably, the β-lactamase activity (Figure 1c) and the amount (Figure 1d) of OMVs produced by NTHi at the time of its isolation from the patient’s tonsils (reflecting the situation in vivo during therapy) significantly decreased when the isolate was passaged in vitro in a medium without amoxicillin, and significantly increased when amoxicillin in concentrations reported in the tonsillar tissue during amoxicillin treatment (0.17 µg/mL to 3.9 µg/mL) [30,31,32] was added to the NTHi culture (Figure 1c,d). Thus, amoxicillin in concentrations reached in the tonsils during therapy significantly increased secretion and enzymatic activity of NTHi β-lactamase-containing OMVs. This led us to hypothesize that these β-lactamase-containing, amoxicillin-induced OMVs (hereafter termed NTHi OMVs_βL+AMX+_) were involved in the amoxicillin failure to eradicate GAS from the patient by protecting GAS against amoxicillin. 

### 3.3. NTHi OMVs_βL+AMX+_ Protect GAS against Bactericidal Concentrations of Amoxicillin

**Figure 2:**
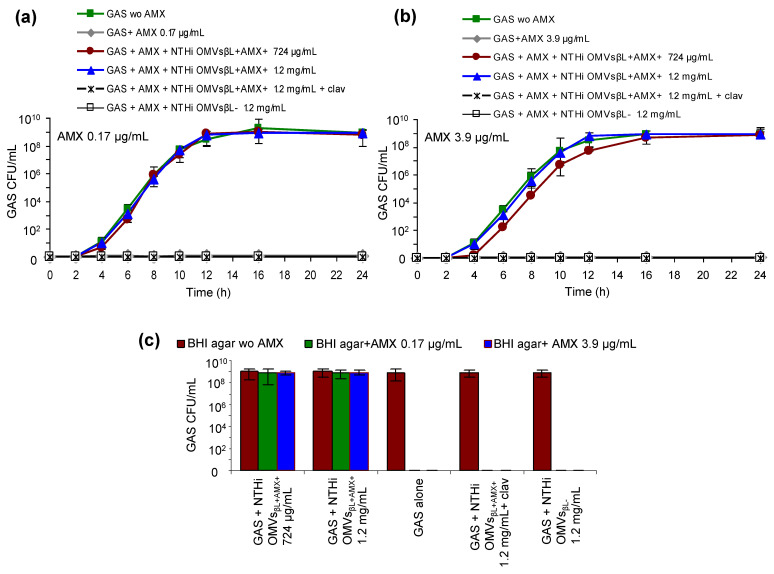
Amoxicillin-induced, β-lactamase-carrying OMVs from amoxicillin-resistant nontypeable *Haemophilus influenzae* (NTHi OMVs_βL+AMX+_) protect GAS against bactericidal concentrations of amoxicillin: (**a**,**b**) GAS growth in BHI broth without amoxicillin or with amoxicillin 0.17 µg/mL (**a**) or 3.9 µg/mL (**b**) in the absence or presence of the indicated amounts of NTHi OMVs_βL+AMX+_ without or with clavulanate, or in the presence of control β-lactamase-negative NTHi OMVs (NTHi OMVs_βL−_). Data are means ± standard deviations (SDs) from three independent experiments. (**c**) Growth of GAS from 24 h cultures shown in (**a**,**b**) on BHI agar without or with amoxicillin. Data are means ± SDs from three independent experiments.

To test the hypothesis that NTHi OMVs_βL+AMX+_ are involved in the amoxicillin failure to eradicate GAS from the patient, we determined whether these OMVs protect GAS against the reported amoxicillin tonsillar concentrations of 0.17 µg/mL and 3.9 µg/mL [30,32], which represent ~10-fold and 244-fold MICs, respectively, for this isolate (MIC of 0.016 µg/mL) (Table 2). To this end, GAS growth was monitored for 24 h in the presence of each amoxicillin concentration and NTHi OMVs_βL+AMX+_ in the doses of 724 µg/mL or 1.2 mg/mL, which were induced by the respective amoxicillin concentrations (Table 1). Indeed, each NTHi OMVs_βL+AMX+_ dose protected GAS against amoxicillin, with the protection being slightly delayed with 724 µg/mL of NTHi OMVs_βL+AMX+_ against 3.9 µg/mL of amoxicillin (Figure 2a,b). This demonstrated that NTHi OMVs_βL+AMX+_ in the amounts induced by amoxicillin concentrations reached in the tonsils during therapy protected GAS against bactericidal effect of amoxicillin. No GAS protection was conferred by β-lactamase-negative OMVs from a control amoxicillin-susceptible NTHi (Figure 2a,b), indicating that the β-lactamase associated with NTHi OMVs_βL+AMX+_ was the GAS-protecting component.

### 3.4. NTHi OMVs_βL+AMX+_ Increase Amoxicillin MIC for GAS

To elucidate the basis for the NTHi OMVs_βL+AMX+_-mediated GAS protection against amoxicillin, we determined the effect of these OMVs on amoxicillin MIC for GAS. We found that in the presence of 724 µg/mL and 1.2 mg/mL of NTHi OMVs_βL+AMX+_, the amoxicillin MIC for GAS increased from 0.016 µg/mL to 4 µg/mL (250-fold) and to 16 µg/mL (1000-fold), respectively (Table 3), making GAS resistant to amoxicillin. Importantly, both the amoxicillin MIC increase for GAS and the GAS protection against amoxicillin via NTHi OMVs_βL+AMX+_ were inhibited by the β-lactamase inhibitor clavulanate (Figure 2a,b, Table 3), confirming that these effects were mediated by the NTHi OMVs_BL+AMX+_-associated β-lactamase. Moreover, GAS failed to grow on a medium with amoxicillin in the absence of NTHi OMVs_βL+AMX+_ (Figure 2c), demonstrating that these OMVs, not acquisition of the *bla*_TEM-1_ gene from NTHi, accounted for its amoxicillin resistance. The absence of *bla*_TEM-1_ in GAS was confirmed by PCR (Table 2).

### 3.5. NTHi β-Lactamase-Carrying OMVs Are Secreted In Vivo at the Infection Site

**Figure 3:**
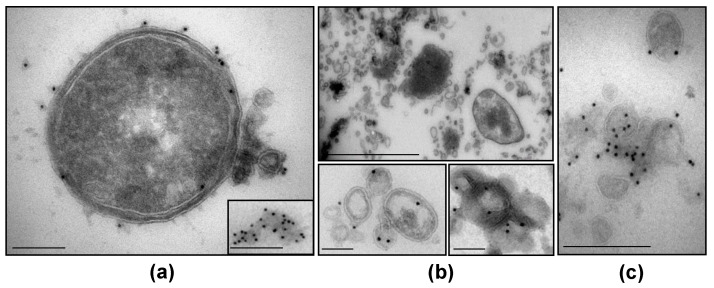
Transmission electron microscopy of the patient’s pharyngotonsillar swab, crypt exudate, and saliva demonstrating in vivo secretion of β-lactamase-carrying NTHi OMVs: (**a**) NTHi secreting β-lactamase-carrying OMVs and released β-lactamase-carrying OMVs (inset) in pharyngotonsillar swab. (**b**) NTHi bacteria surrounded by large amounts of OMVs (top) and released β-lactamase-carrying OMVs (bottom) in tonsillar crypt exudate. (**c**) β-lactamase-carrying OMVs in saliva. Pictures are ultrathin cryosections stained (except for (**b**) top which is unstained) with anti-β-lactamase antibody and 10 nm gold-conjugated secondary antibody. Scale bars: (**a**,**c**), 200 nm; (**b**) top, 1 µm, bottom, 100 nm.

To provide a final piece of evidence for the involvement of NTHi OMVs_βL+AMX+_ in the amoxicillin therapy failure, we searched for their secretion in vivo at the site of infection. We identified NTHi bacteria secreting β-lactamase-carrying OMVs as well as released, free β-lactamase-carrying OMVs in the patient’s pharyngotonsillar swabs (Figure 3a), tonsillar crypt exudate (Figure 3b), and saliva (Figure 3c). Taken together, our findings demonstrate that amoxicillin-resistant NTHi colonizing the pharyngotonsillar mucosa of the GAS-infected patient secreted in situ β-lactamase-carrying OMVs, which were inducible by amoxicillin and protected GAS against the antibiotic, thereby accounting for the therapy failure. 

## 4. Discussion

This study brings a new insight into the mechanisms of amoxicillin therapy failure in patients with GAS pharyngotonsillitis. The involvement of β-lactamase-carrying OMVs secreted by NTHi colonizing the patient’s pharynx and tonsils in this failure is supported: (i) by the induction of NTHi OMV secretion and OMV-associated β-lactamase activity by amoxicillin concentrations reached in the tonsils during therapy; (ii) by the ability of NTHi OMVs_βL+AMX+_ to significantly increase amoxicillin MIC for GAS and to protect GAS against bactericidal concentrations of amoxicillin; (iii) by the inhibition of each of these NTHi OMVs_βL+AMX+_-mediated effects by the β-lactamase inhibitor clavulanate; (iv) by the inability of GAS to resist amoxicillin in the absence of NTHi OMVs_βL+AMX+_, which is in accordance with its excellent susceptibility to amoxicillin [1]; and (v) by the secretion of NTHi OMVs_βL+AMX+_ in vivo at the site of infection. This is, to the best of our knowledge, the first evidence that OMV-mediated protection of bacteria against β-lactam antibiotics previously observed under in vitro conditions [18,22,23,24,25,26] has a clinical parallel in the ability of β-lactamase-carrying OMVs secreted in vivo to protect pathogens against β-lactams during patients´ treatment, thus leading to therapy failure. For patients with GAS pharyngotonsillitis, this mechanism may be of a particular importance, since more than one fourth of children with GAS pharyngotonsillitis have their tonsils colonized with *H. influenzae* or *M. catarrhalis* [37], both of which can produce β-lactamase-carrying OMVs ([22,23], this study). Mechanistically, the structural identity between the bacterial outer membrane and OMV membrane allows β-lactams to enter, through the porin channels, the OMV lumen, where β-lactamase, originating from the bacterial periplasm, is located and hydrolyzes the antibiotics [22,23,24,38]. Through this process, OMVs secreted outside bacterial cells serve as a first line of protection against β-lactams before the antibiotics reach the target bacterial population. Since OMVs secreted by Gram-negative β-lactam-resistant bacteria in vitro carry a broad spectrum of β-lactamases [18,22,23,24,25,26], it is likely that a similar mechanism that we have described for amoxicillin plays a role in therapeutic failures of other β-lactam antibiotics including carbapenems, which are the “last resort” β-lactams used to combat multidrug resistant pathogens [26]. Moreover, experimental data suggest that OMVs may also be involved in antimicrobial resistance in other ways, including the sequestration of membrane active antibiotics (polymyxin B, colistin) [8,20,21,39] and dissemination of antibiotic resistance genes [33,40,41]. Thus, OMVs may serve as universal bacterial tools contributing, by different mechanisms, to antibiotic resistance. A broad involvement of OMVs in antimicrobial resistance is strongly supported by the observations that the increase in OMV secretion (e.g., by hypervesiculating mutans) increased the resistance, and the reduction in or inhibition of OMV secretion increased the susceptibility of various bacteria to a range of antibiotics [21,39,42]. 

The mechanism by which amoxicillin (and other β-lactam antibiotics such as imipenem [17] and meropenem [16]) increases OMV production is presently not known. Based on the models of OMV biogenesis [6] and the mechanism of action of β-lactam antibiotics [43], we hypothesize that the inhibition of peptidoglycan polymerization due to the β-lactam binding to the penicillin-binding proteins [43] plays a key role. This hypothesis is supported by the occurrence of OMV budding at the sites of locally decreased crosslinking between the peptidoglycan and the outer membrane [6]; it is in accordance with the peptidoglycan being a central structure that accounts, via its crosslinks with various membrane proteins, for the stability of the bacterial envelope [6]. 

The therapeutic success of amoxicillin/clavulanate in our patient demonstrated that amoxicillin’s failure to eradicate GAS was not due to a poor tonsillar penetration of the drug. However, the amoxicillin concentration in the patient’s tonsils could not be determined, as he did not undergo a tonsillectomy. This is the reason why we used the concentrations reported in the tonsils of amoxicillin-treated patients who did undergo a tonsillectomy [30,31,32] to determine the effects of amoxicillin on the amount of OMVs produced by NTHi and the OMV β-lactamase activity. Since these concentrations encompass a broad range (0.17 µg/mL–3.9 µg/mL), it is likely that the amoxicillin concentration in the tonsils of our patient was within this range. To further evaluate the mechanism of β-lactam therapy failure reported in this study in other clinically relevant situations, we will continue our investigations in additional patients with pharyngotonsillitis, in whom a coinfection with β-lactam-susceptible (GAS and others) and beta-lactam-resistant bacteria is detected, and the β-lactam antibiotic therapy failure occurs. 

## 5. Conclusions

The pathogen protection via β-lactamase-carrying OMVs secreted in situ by the flora coinhabiting the infection site represents a yet underestimated mechanism of β-lactam therapy failure. The extent of the involvement of this mechanism in β-lactam therapy failure in clinical praxis needs to be evaluated in further clinical–microbiological studies. The emerging role of OMVs in antibiotic resistance should be taken into account in strategies directed at combating this serious medical and public health problem. 

## Figures and Tables

**Table 1:** Effect of amoxicillin on protein concentrations of OMVs produced by NTHi patient’s isolate.

NTHi OMVs Produced under Conditions	OMV Protein Concentration (µg/mL)
BHI broth without amoxicillin	325
BHI broth + amoxicillin 0.17 µg/mL ^1^	724
BHI broth + amoxicillin 1.1 µg/mL ^2^	989
BHI broth + amoxicillin 3.9 µg/mL ^3^	1200

NTHi, nontypeable *Haemophilus influenzae*; OMVs, outer membrane vesicles; BHI, brain heart infusion. ^1,2,3^ Amoxicillin concentrations reported in the tonsils of amoxicillin-treated patients who underwent tonsillectomy [30,31,32].

**Table 2:** Antimicrobial susceptibilities of GAS and NTHi isolated from the patient’s throat culture.

Isolate	Disc Diffusion Method		Broth Microdilution Method MIC (µg/mL)		PCR *bla*_TEM-1_
	Susceptible to	Resistant to	Amoxicillin	Amoxicillin/ Clavulanate	
GAS ^1^	Penicillin, Ampicillin Amoxicillin	None of tested	0.016	0.016	Negative
NTHi	Amoxicillin/ clavulanate	Ampicillin Amoxicillin	16	0.5	Positive

GAS, *Streptococcus pyogenes* group A; NTHi, nontypeable *Haemophilus influenzae*. ^1^ The data refer to the GAS isolates obtained before amoxicillin treatment and after amoxicillin treatment.

**Table 3:** The influence of NTHi OMVs_βL+AMX+_ on amoxicillin MIC for GAS isolated from the patient’s throat culture.

GAS Culture Tested for Amoxicillin MIC	Amoxicillin MIC for GAS (µg/mL)
GAS alone	0.016
GAS + NTHi OMVs_βL+AMX+_^1^ (724 µg/mL)	4
GAS + NTHi OMVs_βL+AMX+_ (1.2 mg/mL)	16
GAS + NTHi OMVs_βL+AMX+_ (1.2 mg/mL) + clavulanate	0.032
GAS + NTHi OMVs_βL−_^2^ (1.2 mg/mL)	0.016

GAS, *Streptococcus pyogenes* group A; MIC, minimal inhibitory concentration; NTHi, nontypeable *Haemophilus influenzae*. ^1^ NTHi OMVs_βL+AMX+_, β-lactamase-carrying, amoxicillin-induced OMVs from NTHi patient’s isolate. ^2^ NTHi OMVs_βL−_, β-lactamase-negative OMVs from a control amoxicillin-susceptible NTHi.

## Data Availability

The datasets supporting the results of this article are included within the article.
